# Impact of Chronic Cold Water Immersion and Vitamin D3 Supplementation on the Hippocampal Metabolism and Oxidative Stress in Rats

**DOI:** 10.3390/cells14090641

**Published:** 2025-04-26

**Authors:** Daria Korewo-Labelle, Mateusz Jakub Karnia, Dorota Myślińska, Jan Jacek Kaczor

**Affiliations:** 1 Department of Physiology, Faculty of Medicine, Medical University of Gdansk, 80-211 Gdansk, Poland; daria.korewo-labelle@gumed.edu.pl; 2 Department of Animal and Human Physiology, Faculty of Biology, University of Gdansk, 80-308 Gdansk, Poland; mateusz.karnia@ug.edu.pl (M.J.K.); dorota.myslinska@ug.edu.pl (D.M.)

**Keywords:** cold stress, vitamin D3, neuroprotection, mitochondrial dysfunction, hippocampus

## Abstract

Chronic cold exposure is a stressor that may adversely affect the hippocampal structure and cognitive function. Critical for memory formation and learning processes, the hippocampus is particularly susceptible to hypothalamic–pituitary–adrenal (HPA) axis activity and elevated glucocorticoid levels. Vitamin D plays a complex role in regulating mitochondrial function and may provide neuroprotection. This study aimed to investigate the effects of chronic cold exposure on proteins associated with signaling pathways, mitochondrial function, and oxidative stress in the hippocampus of rats and to evaluate the neuroprotective potential of vitamin D3 supplementation. Male Wistar rats (n = 26) were assigned to four groups: control (CON; n = 4), sham stress (WW; n = 6), chronic cold water immersion (CCWI) (CW group; n = 8), and CCWI with 600 IU/kg/day vitamin D3 (VD3) supplementation (CW + D group; n = 8). Exposure to CCWI significantly reduced the hippocampal mass of rats, an effect not reversed by vitamin D3 supplementation. However, vitamin D3 improved mitochondrial function and exhibited antioxidant effects, partially reducing markers of protein and lipid free radicals damage in neural tissue. Our findings demonstrate the antioxidant properties of VD3 and its potential role in mitigating hippocampal damage during prolonged cold exposure, although its neuroprotective effects remain limited.

## 1. Introduction

Environmental factors, such as changes in temperature, strongly affect the body’s response. Activation of the thermoregulatory center in the hypothalamus, along with the hypothalamic–pituitary–adrenal (HPA) axis and the locus coeruleus–norepinephrine system, triggers a cascade of adaptive mechanisms that enable the body to manage low temperatures [1,2]. Empirical evidence suggests that acute and prolonged exposure to cold increases the activity of the HPA axis, leading to increased release of corticosterone (CORT) into the bloodstream of rodents [3,4].

The hippocampus, as a structure of the limbic system, plays a central role in memory formation, spatial processing, neuroplasticity, and the stress response. However, it is particularly susceptible to damage. Glucocorticosteroids (GC) may disrupt the HPA axis and impair hippocampal function by binding to the glucocorticoid (GR) and mineralocorticoid (MR) receptors, which are densely concentrated in this structure [5]. Under physiological conditions, MR and GR regulate many neuronal differentiation and excitability processes. MR is essential for memory evaluation and recall and is activated during the early stress phase. At the same time, GR activation is crucial for memory consolidation and behavioral adaptation [6].

It is concluded that high levels of GC may contribute to energy metabolism dysfunction and dysregulate redox stress, and any changes in metabolism promote metabolic deficits in neurons. Due to the brain’s high energy demands, such deviations in metabolism may lead to neuronal dysfunction [7]. Consequently, prolonged exposure to the GC significantly reduces the volume of the hippocampus and decreases its neurogenesis and synaptic plasticity [8].

To apply the acute stress model, in vivo studies use a single cold exposure, while multiple and repeated exposures are used to induce a chronic stress response [9]. More recently, results have shown that cold exposure may induce corticosteroid-induced oxidative stress and activate the apoptotic pathway in hippocampal neurons [10]. Although exposure of the body to low environmental temperatures is a common stressor, it can provide many health benefits under certain conditions. Several studies suggest that cold water bathing reduces mood disorders [11], supports endocrine function [12], and the immune system [13]. However, studies related to chronic cold exposure are inconclusive. Some suggest a link to the adaptive mechanisms of the HPA axis and/or a lack of effect on memory processes [4], while others suggest that cold exposure is a potent stressor. Prolonged cold exposure stress has been shown to cause neuroinflammation induced by microglia activation and damage to hippocampal neurons [14]. Another study shows that it causes neuronal loss in the CA1 and CA3 regions of the hippocampus [15]. Therefore, cold stress may contribute to neuronal damage through the disruption of key proteins involved in neuronal survival and plasticity, such as neurofilament light chain (NFL) and RNA-binding protein 3 (RBM3), a cold shock protein.

In recent years, the topic of vitamin D has been one of the main neurosteroids involved in neurogenesis and neuroplasticity in the brain. The action of vitamin D is not limited to the regulation of calcium–phosphate metabolism but exhibits pleiotropic activity [16]. Severe vitamin D deficiency has been linked to various diseases, including psoriasis, multiple sclerosis, rheumatoid arthritis, and respiratory infections. Moreover, low serum vitamin D concentration is associated with mental health disorders like schizophrenia and depression, as well as neurodegenerative diseases [17]. Recently, it has been shown that supplementation with vitamin D3 stimulates the vitamin D receptor (VDR), which potentially contributes to neuroprotection in dementia and neurodegenerative diseases [18].

Mitochondria are critical for maintaining neuronal energy homeostasis and facilitating adaptive responses to stress. However, chronic stress exposure may impair mitochondrial biogenesis, affecting ATP production and overall mitochondrial function. Vitamin D may modulate neuroinflammation, mitochondrial biogenesis and function, and oxidative stress by regulating *VDR* expression and protein content [19]. Despite these insights, the extent to which chronic cold water immersion (CCWI) alters specific receptors and affects hippocampal function remains unclear. The present study employs a 28-day CCWI protocol, which—according to the available literature—has not been previously applied in this form. The extended duration of exposure was intended to reproduce a state of chronic environmental stress with low daily intensity, allowing for the evaluation of its cumulative effect on hippocampal tissue. Given the established susceptibility of this structure to prolonged HPA axis activation, this study aimed to investigate the impact of CCWI on hippocampal proteins involved in signaling pathways, mitochondrial function, and oxidative stress and to assess the neuroprotective potential of vitamin D3 supplementation in mitigating these effects.

## 2. Materials and Methods

### 2.1. Animals and Experiment Protocol

Male Wistar rats were obtained from the Academic Experimental Animal House at the Medical University of Gdansk, Poland. When the experiment began, animals aged three months and weight 300–400 g were randomly divided into four groups: (I) control (CON, n = 4), (II) sham stressed by immersion in warm water (WW, n = 6), (III) chronic immersed in cold water receiving an oral placebo (vegetable oil; CW, n = 8), and (IV) chronic immersed in cold water supplemented with vitamin D3 (600 IU/kg Juvit D3; CW + D, n = 8). For twenty-eight days of the experiment, the animals were placed individually in a glass tank measuring 21 × 15 × 30 cm filled with water to a depth of 1 cm, where they spent 60 min/day. In the CW and CW + D groups, the temperature of the ice water was around 4 °C, while in the WW group, it was 34–36 °C. Each day of the experiment, animals in the CW + D group received 1–1.5 drops of vitamin D3 into their mouths, and animals in the CW group received a placebo by soft oral pipette administration without rigid sondage. Under controlled laboratory conditions (temperature: 22 ± 2 °C and humidity: 55 ± 2%), the animals were housed in groups of 3–4 per cage. They were kept on a 12:12 h light/dark cycle and had free, non-limited access to food and water. All animal studies were performed with the Ethics Committee Approval (No. 10/2019). After twenty-eight days of the experiment, the animals were anesthetized using isoflurane inhalation anesthesia (2.5%, flow rate 0.5 L/min). Animals were then euthanized by decapitation. The rat hippocampus was isolated post-mortem, weighed, and frozen by placing it in liquid nitrogen. The material was stored at −80 °C in microtubes for further biochemical analyses.

### 2.2. Immunoblot Analysis

Hippocampus was homogenized in RIPA buffer (Pierce RIPA Buffer, 89901, Thermo Scientific, Waltham, MA, USA) with the addition of protease and phosphatase inhibitors 100X Halt™ Protease and Phosphatase Inhibitor Cocktail (1861280, Thermo Scientific, Waltham, MA, USA) in a ratio of 1:100. The 8% homogenate was centrifuged at 750× *g*, then collected supernatant was centrifuged at 12,000× *g* in 4 °C. Supernatants containing 25–30 μg of protein were separated by electrophoresis in mini-PAGE gels (10% or 12%) using ready-to-use TGX Precast Protein Gels (4561035, 4561045; BioRad, Hercules, CA, USA). Electrophoresis was performed under 125 V, 0.05 A, 6 W conditions for about 50 min. Then, proteins were transferred to the membrane by the electrotransfer method for 7 min in the “Trans-Blot Turbo Transfer System” (BioRad, Hercules, CA, USA) at 25 V. Membranes were blocked for 30 min in “Every Blot Blocking Buffer” (12010020; BioRad, Hercules, CA, USA) or for 2 h in 5% skimmed milk in TBST. The incubation took place overnight with the primary antibodies listed in Table 1. After washing five times in TBST, the membranes were incubated for one hour with HRP-conjugated secondary antibodies listed in Table 1.

Results were visualized using the “Clarity Western ECL Substrate” kit (1705061; BioRad, Hercules, CA, USA) and recorded using the “ChemiDoc MP” system (BioRad, Hercules, CA, USA). The results obtained were analyzed using the “Image Lab 6.1” software (BioRad, Hercules, CA, USA). Data were normalized against the total protein content of the sample, and values were expressed relative to the respective controls.

### 2.3. Determination of Markers of Free Radical Damage

#### 2.3.1. Determination of the Content of Thiol (-SH) Groups

The rest of the hippocampi were homogenized in buffer containing 50 mM Tris-HCl, 150 mM NaCl, 1 mM EDTA, 0.5 mM DTT pH 7.2, and 0.2% protease inhibitors cocktail (P834; Sigma-Aldrich, St. Louis, MO, USA). The 4% tissue homogenates were centrifuged at 750× *g* for 10 min at 4 °C. The supernatant was frozen and stored at −80 °C for further analysis. According to the manufacturer’s instructions, protein concentration in hippocampal supernatant was assessed using the Pierce BCA Protein Assay Kit (23225, Thermo Scientific, Waltham, MA, USA).

Determination of the -SH groups, one of the markers of free radical damage of proteins, was performed as follows: 200 µL of 10 mM PBS buffer (pH 8.0) was added to 20 µL of 750× *g* supernatant. To expose the -SH groups of the proteins, 30 µL of 10% sodium dodecyl sulfate and 30 µL of 0.1 mM 2,2-dithiobisnitrobenzoic acid (DTNB) were added. The whole mixture was incubated for 1 h at 37 °C. Absorbance was measured with a microplate reader (Varioskan Flash—Spectral Scanning Multimode Microplate Reader 183, Thermo Scientific, Waltham, MA, USA) at a wavelength of 405 nm and a temperature of 37 °C. Values for -SH groups were calculated relative to the blank (without DTNB) from a standard curve for reduced glutathione (GSH; GA251-10G, Sigma-Aldrich, St. Louis, MO, USA). The concentration of -SH groups was expressed in µmol/g tissue and µmol/mg of protein.

#### 2.3.2. Determination of Malondialdehyde (MDA) Levels

Free radical lipid damage in the hippocampus was determined by measuring malondialdehyde (MDA) concentration. Lysates containing 0.05% butylhydroxytoluene (BHT) were centrifuged at 4000× *g* at 4 °C for 10 min. Then, 100 µL of samples, and 75 µL of 37% HCl were added to 325 µL of R1 reaction solution (0.064 g of 1-methyl-2-phenylindole into 30 mL of acetonitrile to which 10 mL of methanol was added to bring the volume to 40 mL). After an hour-long incubation at 45 °C, the reaction was stopped for 1 min by cooling the samples in a container of ice. The samples were centrifuged at 5500 RPM for 10 min, and supernatant was collected. Absorbance was measured at 586 nm. The concentration of MDA in the samples was determined using 10 mM 1,1,3,3-tetramethoxypropane as a standard. MDA concentration was expressed in nmol/g tissue.

#### 2.3.3. Determination of 8-Isoprostanes (8-iso) Levels

An enzymatic immunoassay ELISA analyzing 8-isoprostanes (516351; Cayman Chemical; Ann Arbor, MI, USA) in the hippocampal lysate was used to assess free radical-induced lipid damage. According to the manufacturer’s recommendations, the material for analysis contained a 0.05% BHT solution. The absorbance of the samples was measured at 405 nm and 37 °C. Measurement results were expressed in pg/mL of sample.

### 2.4. Enzymatic Activity Measurements

Citrate synthase (CS) activity was performed according to the procedure outlined in Korewo-Labelle et al. [20]. In brief, 173 μL of buffer (50 mM TRIS-HCl, 5 mM EDTA; pH 8.1) was added to each well of a 96-well plate, followed by the addition of 3 μL of 750× *g* supernatant. Next, 20 μL of freshly prepared 1 mM DTNB, 2 μL of 15 mM acetyl-coenzyme A, and 2 μL of 10 mM oxaloacetic acid were added to the mixture. CS activity was measured in duplicates for two minutes using a microplate reader (Varioskan Flash—Spectral Scanning Multimode Microplate Reader 183, Thermo Scientific, Waltham, MA, USA) at an absorbance of 412 nm and a temperature of 37 °C. The activity was expressed in μmol/min/mg protein.

### 2.5. Statistical Analysis

Data analysis was conducted using the statistical software GraphPad Prism 8.3 (GraphPad Software, Boston, MA, USA). A one-way ANOVA was conducted to evaluate the hippocampal mass, protein content, enzyme kinetics, and immunoassay results, followed by a post hoc test, the Least Significant Difference (LSD). The results are the mean ± standard deviation (SD), with statistical significance determined at *p* ≤ 0.05.

## 3. Results

**Figure 1:**
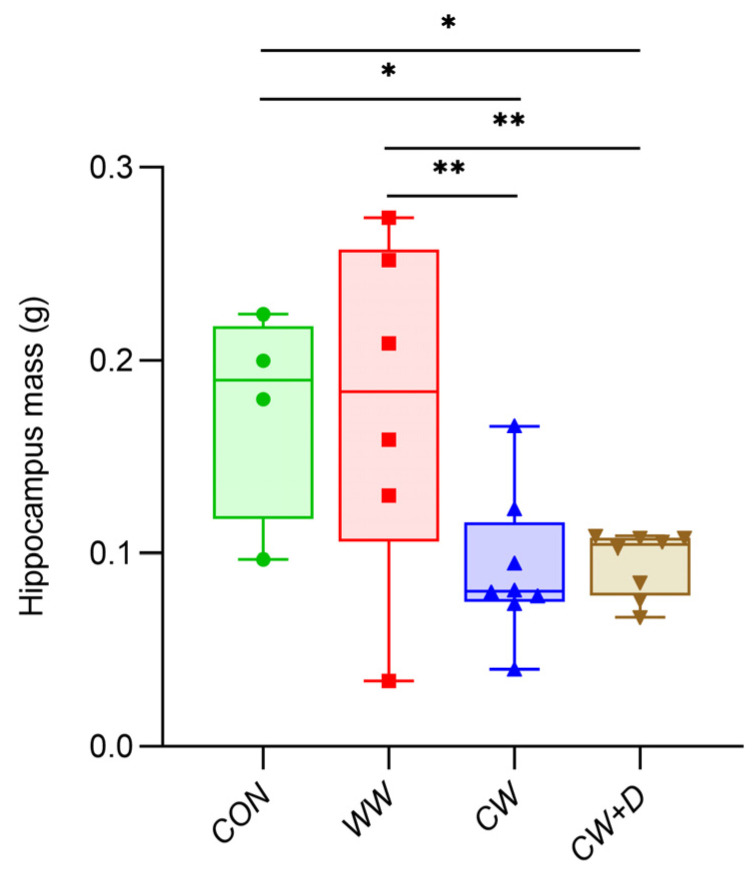
The hippocampus mass of rats from CON (n = 4), WW (n = 6), CW (n = 8), and CW + D (n = 8) groups. All data are presented as mean ± SD; * *p* < 0.05; ** *p* < 0.01.

CCWI led to a significant reduction in hippocampal mass, with the CW (0.09 ± 0.04 g) being lower than the CON (0.18 ± 0.06 g; * *p* < 0.05) and the WW (0.18 ± 0.09 g; ** *p* < 0.01). In the CW + D group, the hippocampal mass was also lower (0.10 ± 0.02 g) than in the CON and WW groups (* *p* < 0.05 and ** *p* < 0.01, respectively; Figure 1).

**Figure 2:**
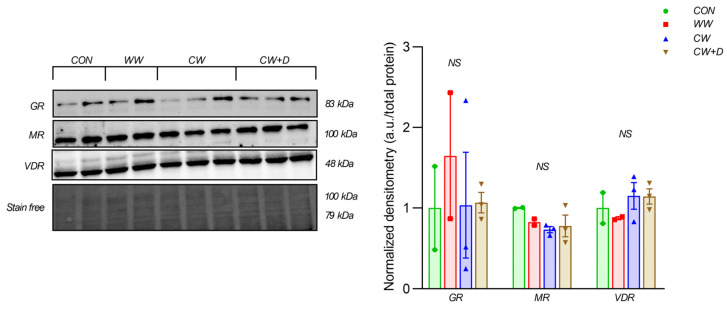
The content of specific receptors (GR, MR, and VDR) in the hippocampus of rats in the CON (n = 2), WW (n = 2), exposure to CCWI (CW; n = 3), and supplemented with vitamin D3 during CCWI (CW + D; n = 3). All data are presented as mean ± SD. NS—non-significant. The results were normalized to the total protein content.

CCWI partially influenced stress-related proteins. GR content was highest in the WW (1.65 ± 1.10), followed by the CW + D (1.07 ± 0.22) and the CW (1.03 ± 1.14), while the CON (1.00 ± 0.73) had the lowest levels. MR content was reduced in the CW (0.73 ± 0.07) compared to the CON (1.00 ± 0.01) and the WW (0.83 ± 0.06), and supplementation in the CW + D group (0.78 ± 0.23) showed no full recovery. VDR content was higher in the CW (1.15 ± 0.29) compared to the CON (1.00 ± 0.27) and the WW (0.87 ± 0.03). This elevation persisted in the CW + D group (1.14 ± 0.16), suggesting a response to vitamin D supplementation (Figure 2). Although the protein content of the above receptors was observed, no statistically significant differences were noted.

**Figure 3:**
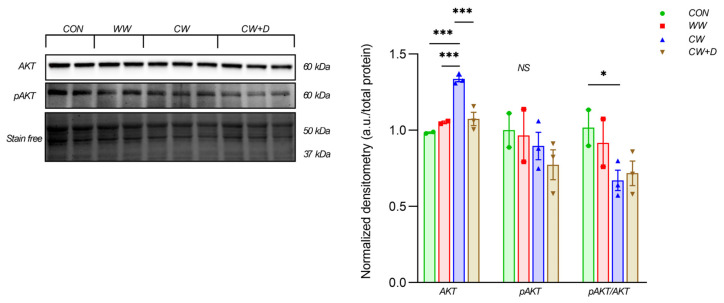
The protein content of AKT, pAKT, and the pAKT/AKT ratio in rat hippocampus in CON (n = 2) and WW (n = 2) groups and treated with CCWI (CW; n = 3) as well as supplemented with vitamin D3 (CW + D; n = 3). All data are presented as mean ± SD; * *p* < 0.05, *** *p* < 0.001, NS—non-significant. The results were normalized to the total protein content.

Proteins involved in cellular survival and metabolism were also affected. AKT content was significantly higher in the CW (1.34 ± 0.03) compared to the CON (0.98 ± 0.01; *** *p* < 0.001), WW (1.05 ± 0.01; *** *p* < 0.001), and CW + D (1.07 ± 0.08; *** *p* < 0.001). However, pAKT was lower in the CW (0.90 ± 0.16) than in the CON (1.00 ± 0.16) and the WW (0.97 ± 0.24). In the CW + D, pAKT was further reduced (0.77 ± 0.17). The pAKT/AKT ratio was lowest in the CW (0.67 ± 0.12) compared to CON (1.02 ± 0.17; * *p* < 0.05) and the WW (0.92 ± 0.22), with the CW + D (0.72 ± 0.14) showing a slight increase from the CW but not restored to control levels (Figure 3).

**Figure 4:**
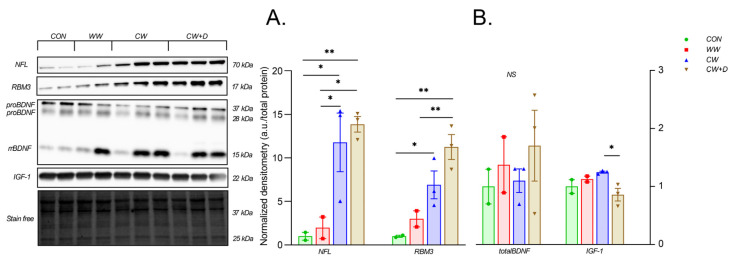
Western blot of NFL and RBM3 (**A**) and IGF-1 and total BDNF (**B**) in the hippocampus in the CON (n = 2), WW (n = 2), and after CCWI exposure (CW; n = 3) and supplemented with vitamin D3 (CW + D; n = 3). All data are presented as mean ± SD; * *p* < 0.05; ** *p* < 0.01, NS—non-significant. The results were normalized to the total protein content.

CCWI affects the content of proteins related to stress response, mitochondrial function, and neuronal integrity. NFL, a marker of neuronal stress, was significantly elevated in the CW (11.79 ± 5.85) compared to the CON (1.00 ± 0.65; * *p* < 0.05) and WW (1.97 ± 1.77; * *p* < 0.05). In the CW + D, NFL content (13.87 ± 1.57) was slightly lower than in CW but remained elevated, indicating sustained neuronal stress or damage despite supplementation. Also, the NFL was significantly elevated compared to the CON and the WW groups (** *p* < 0.01 and * *p* < 0.05, respectively; Figure 4A). RBM3, a cold shock protein, was strongly upregulated in CW (6.91 ± 2.77) compared to CON (1.00 ± 0.18; * *p* < 0.05) and WW (3.00 ± 1.30). Supplementation in CW + D (11.26 ± 2.49) further increased RBM3 levels to 11.25 ± 2.49 a.u. (** *p* < 0.01 vs. the CON and WW groups; Figure 4A). BDNF content was slightly increased in the CW (1.10 ± 0.35) compared to the CON (1.00 ± 0.42), with the CW + D (1.70 ± 1.06) showing the highest levels, however, without statistical significance. IGF-1 content was higher in the CW group (1.25 ± 0.03) compared to the CON (1.00 ± 0.17) and the WW (1.13 ± 0.07). In the CW + D, IGF-1 content was lower (0.86 ± 0.19) compared with the CW (* *p* < 0.05) but remained below the control values (Figure 4B).

**Figure 5:**
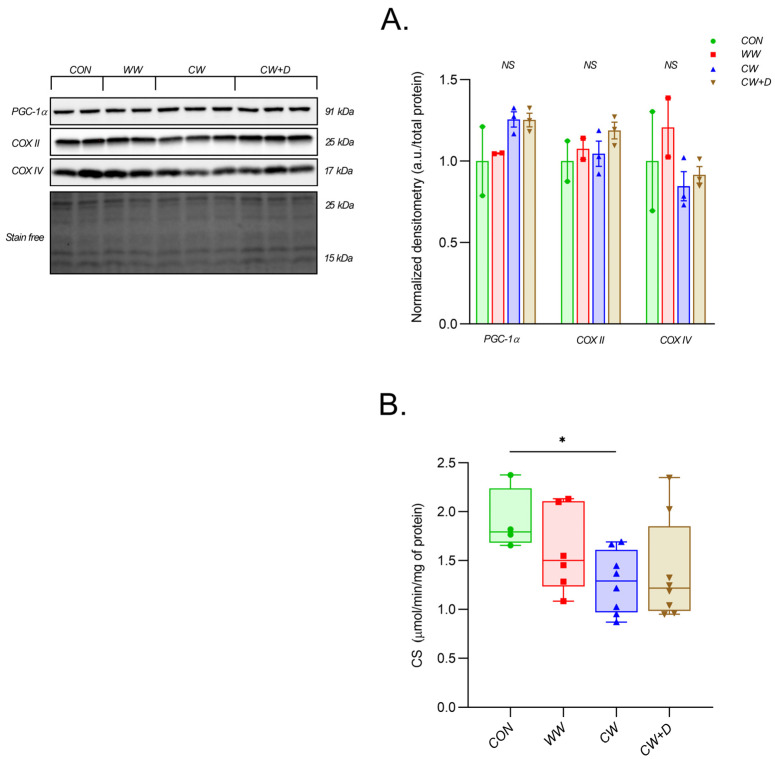
The protein content of PGC-1α, COX II, and COX IV. (**A**) The hippocampus of rats from CON (n = 2) and WW groups (n = 2), as well as after exposure to CCWI (CW; n = 3) and supplemented with vitamin D3 during CCWI (CW + D, n = 3). The activity of CS (**B**) in the CON (n = 4), WW (n = 6), CW (n = 8), and CW + D (n = 8) groups, respectively. All data are presented as mean ± SD; * *p* < 0.05, NS—non-significant. The results were normalized to the total protein content for Western blot analysis. CS activity was expressed as µmol/min/mg of protein.

The mitochondrial subunit of cytochrome c oxidase (COX II) showed no significant changes between groups. The content remained similar across conditions, with the CW (1.05 ± 0.13) being slightly lower than the WW (1.08 ± 0.09) but not significantly different from the CON (1.00 ± 0.18). The CW + D group (1.19 ± 0.09) had the highest COX II content. The mitochondrial subunit of cytochrome c oxidase encoded by the nucleus (COX IV) content was also lower in the CW (0.85 ± 0.15) compared to the CON (1.00 ± 0.43) and the WW (1.21 ± 0.26). The CW + D group (0.92 ± 0.09) showed partial restoration but did not return to control levels. Similarly, peroxisome proliferator-activated receptor γ coactivator 1α (PGC-1α), a regulator of mitochondrial biogenesis, was lower in the CW (1.26 ± 0.08) compared to the CON (1.00 ± 0.30) and the WW (1.05 ± 0.00), with the CW + D (1.25 ± 0.07) remaining slightly above the CW. However, it is not significantly different from the CON (Figure 5A). CCWI also affected mitochondrial enzyme activity. CS activity was significantly lower in the CW (1.28 ± 0.32 μmol/min/mg protein) compared to the CON (1.90 ± 0.32 μmol/min/mg protein; * *p* < 0.05) and the WW (1.60 ± 0.43 μmol/min/mg protein). The CW + D group (1.39 ± 0.52 μmol/min/mg protein) only slightly recovered (Figure 5B).

**Figure 6:**
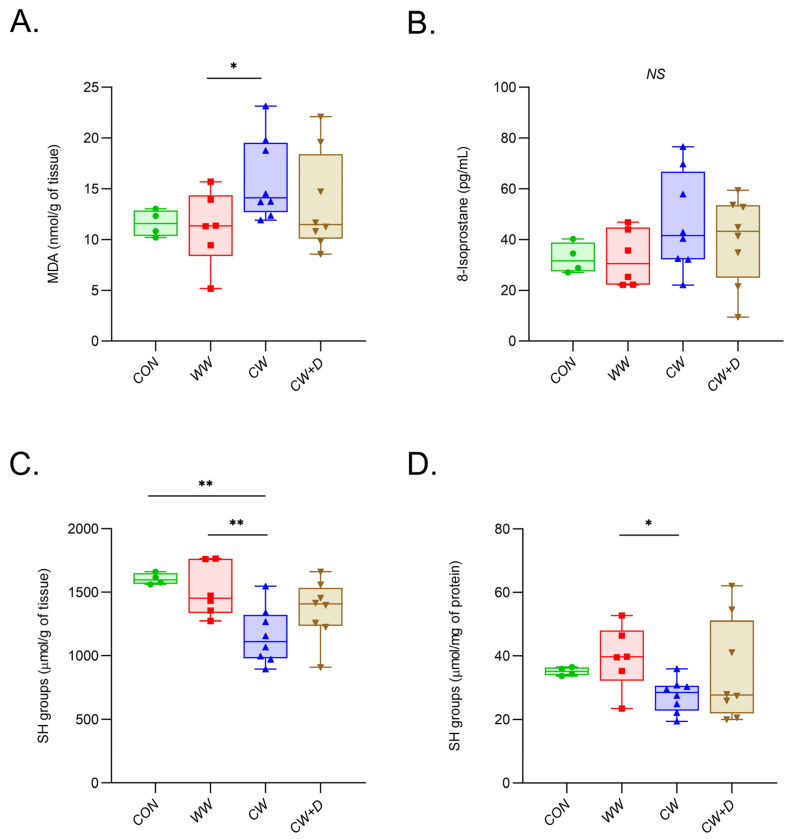
The concentrations of markers of lipid and protein peroxidation. MDA (**A**) and 8-iso (**B**), markers of lipid peroxidation and -SH groups (**C**), and (**D**) marker of protein oxidation in the hippocampus of rats in the CON (n = 4), WW (n = 6), exposure to CCWI (CW; n = 8), and supplemented with vitamin D3 during CCWI (CW + D; n = 8). All data are presented as mean ± SD; * *p* < 0.05; ** *p* < 0.01, NS—non-significant. The results were expressed as nmol/g of tissue (MDA), pg/mL (8-iso). The -SH groups were expressed as µmol/g of tissue (**C**) and µmol/mg of protein (**D**), respectively.

Markers of oxidative stress were altered in response to CCWI. The lipid peroxidation marker, MDA concentration, was significantly higher in the CW (15.99 ± 4.06 nmol/g tissue) compared to the CON (11.60 ± 1.31 nmol/g tissue) and the WW (11.15 ± 3.66 nmol/g tissue; * *p* < 0.05). The CW + D (13.58 ± 4.87 nmol/g tissue) showed a decrease compared to the CW (Figure 6A). Another lipid peroxidation marker, 8-iso concentration was higher in the CW (46.74 ± 19.37 pg/mL) compared to the CON (32.66 ± 6.01 pg/mL) and the WW (32.69 ± 11.07 pg/mL). In the CW + D, 8-iso concentration was lower (39.80 ± 17.14 pg/mL) but not fully restored to control levels (Figure 6B). Cold exposure significantly reduced -SH groups, a marker of protein peroxidation, in the CW (1155.16 ± 218.50 µmol/g tissue) compared to the CON (1605.09 ± 44.94 µmol/g tissue; *p* < 0.01) and the WW (1510.12 ± 206.89 µmol/g tissue; ** *p* < 0.01). In the CW + D, -SH groups were partially restored (1361.38 ± 231.28 µmol/g tissue), and we found no significant differences from the CON and WW groups. When -SH groups were expressed per gram of hippocampus, there still was a statistically significant lower concentration in the CW group compared to both the CON and WW groups (Figure 6C). However, when data were expressed per mg of protein, the direction of changes remained consistent, with significantly reduced -SH groups in the CW (27.60 ± 5.29 µmol/mg of protein) group compared to the WW groups (39.54 ± 9.97 µmol/mg of protein; Figure 6D). These minor differences between groups arise from the reduced and variable protein content in the stress-exposed groups, along with increased intra-group variability, particularly in the CW + D group. Therefore, we believe that normalizing tissue mass may more accurately reflect this model’s overall redox status and oxidative damage.

## 4. Discussion

Our results showed that CCWI disrupts several molecular processes in the hippocampus, including stress signaling, mitochondrial metabolism, and oxidative balance. CCWI induced hippocampal atrophy and reduced CS activity in the rat hippocampus. Additionally, we observed increased oxidative stress, accompanied by elevated markers of free radical-induced damage to lipids and proteins. Moreover, generating reactive oxygen species (ROS) may have inhibited AKT phosphorylation, reducing hippocampal pAKT/AKT ratio after CCWI exposure. Interestingly, the content of NFL and RBM3, proteins involved in neuronal survival, plasticity, and stress adaptation, was significantly elevated during prolonged cold exposure. Although vitamin D3 supplementation did not fully normalize the observed changes, its partial neuroprotective effect was visible across several endpoints. Supplementation with vitamin D3 **conferred partial protection against** oxidative stress and mitochondrial dysfunction and slightly induced a rise in neurotrophic factors in the hippocampus of rats following CCWI.

Recently, we reported that chronic CCWI is a potent stressor. The nearly threefold increase in blood CORT concentration indicates that the HPA axis was partially responsible for the cold shock response [21]. The most notable effect of CCWI in this study was a reduction in MR content. Given MR’s role in maintaining hippocampal excitability and regulating feedback sensitivity to circulating glucocorticoids, this decrease may indicate impaired stress adaptation under prolonged environmental stimulation. Levone et al. [5] showed that prolonged stress lowers MR expression and may interfere with hippocampal plasticity, consistent with our findings.

On the other hand, GR content remained relatively stable, suggesting that MR might be more sensitive to cold exposure in this model. We also noted a tendency toward higher VDR content in both CW and CW + D animals. Through the VDR–ligand complex, vitamin D regulates both genomic pathways and non-genomic signaling via interactions with membrane-associated rapid response steroid binding protein (MARRS) [22] to regulate metabolic respiration and mitochondrial integrity. It is worth noting that previous studies showed VDR upregulation in response to oxidative stress and mitochondrial disturbance [23,24]. The current study observed no statistically significant changes in VDR content following CCWI. This discrepancy may stem from differences in stress-conditioning models. Moreover, as we previously reported, serum vitamin D3 concentration and metabolites remained unchanged after 28 days of CCWI [21]. Therefore, this study’s lack of alterations in VDR content is likely associated with the stability of serum vitamin D3 concentration.

The pAKT/AKT axis was also affected. In the CW group, AKT protein content was elevated. However, both pAKT and the pAKT/AKT ratio were lower, suggesting that the activation of this pathway was inefficient despite the increased total protein content. The chronic stress reduces AKT phosphorylation and weakens neuronal survival [25], which aligns with our observations. Moreover, CCWI may reduce AKT phosphorylation, likely due to elevated CORT, as shown in our previous study [21]. In addition, CCWI reduces the pAKT/AKT ratio via HPA axis overactivation (CORT suppresses AKT) or oxidative stress, as ROS also inhibits AKT phosphorylation, an effect we observed. These changes likely led to hippocampal atrophy, which was detected in this study. In our experiment, pAKT protein content partially bounced back in the CW + D group, which may suggest some involvement of vitamin D3, although additional mechanisms cannot be excluded. Thus, determining the changes in these proteins may provide insight into the neurobiological effects of prolonged cold exposure. NFL content was higher in both the CW and CW + D groups, suggesting that axonal damage or synaptic dysfunction occurred, probably due to compromised structural integrity. Similar findings have been reported before, where increased NFL was linked with a cytoskeletal breakdown during chronic stress [26]. At the same time, we also observed a noticeable rise in RBM3, especially in the CW + D group. A similar observation was made in other models, where cold exposure increased RBM3 expression as part of the adaptive stress response [27]. This parallel increase in NFL and RBM3 reflects the coexistence of degenerative and protective processes in the hippocampus, with vitamin D3 possibly shifting that balance toward repair. RBM3 was upregulated in response to hypothermia and stress in the CW group, and supplementation with vitamin D3 enhanced this effect. Vitamin D3 likely acts as a neuroprotective mechanism by preventing excessive protein degradation, enhancing synaptic plasticity, and promoting neuronal survival without significantly increasing the pAKT/AKT ratio. This is particularly relevant because, while RBM3 typically supports pAKT, elevated RBM3 alone may be insufficient to counteract the metabolic and oxidative stress leading to AKT suppression during CCWI. Therefore, we assume that this phenomenon may represent a form of pathophysiological adaptation to CCWI.

The results showed that calcitriol supplementation increases BDNF content in the hippocampus of rats and alleviates cognitive disorders [28]. The slight, though non-significant, increases in BDNF and IGF-1 content in the CW animals and a trend toward higher BDNF content in CW + D may imply an attempt to restore synaptic plasticity and support neurogenesis. Although IGF-1 content declined in the hippocampus of the CW + D group compared to the CW, BDNF content appeared elevated, potentially indicating a shift in neurotrophic dynamics. Earlier studies reported that vitamin D3 upregulates BDNF expression via the CREB-TrkB-BDNF pathway [29], and the trend observed here may reflect a similar mechanism. In addition, we believe the BDNF to IGF-1 ratio could play a role in the adaptation to chronic stress; however, this hypothesis needs further investigation in future experiments.

Mitochondria are essential for maintaining neuronal energy homeostasis and adaptive responses to stress. Cold exposure affected several mitochondrial markers in our study. While subunit II of cytochrome c oxidase (COX II), encoded by mitochondrial DNA, remained unchanged, the nuclear DNA-encoded subunit IV of cytochrome c oxidase (COX IV) showed a trend toward reduced content in the hippocampus of the CW rats. This may suggest impaired mitochondrial biogenesis and function at the nuclear level. Additionally, citrate synthase (CS) activity was decreased in the CW group, suggesting a disturbance in oxidative metabolism due to CCWI. Surprisingly, the protein content of PGC-1α was not changed significantly. Similar findings were reported in other models of stress-related mitochondrial dysfunction, where lower CS and PGC-1α were associated with energy imbalance [30]. In our study, vitamin D3 supplementation partly reversed these mitochondrial alterations, suggesting a potential role for vitamin D in supporting mitochondrial function through its signaling pathway [31]. Consistent with this, vitamin D3 was shown to protect against ROS generation, enhance *VDR* gene expression and protein content, and improve mitochondrial function [32].

Cold exposure also caused significant oxidative damage in hippocampal tissue. In the CW group, we observed elevated levels of 8-isoprostanes and MDA, indicating lipid peroxidation, and augmented thiol (-SH) groups, demonstrating protein oxidation. These findings align with earlier studies showing increased ROS generation under stress exposure [10,33]. Vitamin D3 supplementation partly reversed some of these changes, especially MDA and -SH group concentrations, which align with its antioxidant role reported before [34]. Although not all markers returned to baseline, our data support the idea that vitamin D3 helps limit oxidative damage in the hippocampus.

Hippocampal mass was lower in cold-exposed rats, and vitamin D3 only partly prevented that loss. The observed changes may result from oxidative stress and impaired mitochondrial function. The issue of inflammatory pathways is also not insignificant. The central anti-inflammatory and antioxidant effects of vitamin D3, which significantly inhibited the expression of TNF-alpha, iNOS, and cyklooxygenase-2 in brain tissue, among others, are well known [35]. The data also indicate that the protective effects of vitamin D3 may extend beyond the realm of structural changes but also involve cognitive function and are related to both promoting antioxidant enzyme activities and immune parameters in the brain [36]. A similar outcome was shown in many chronic glucocorticoid overload states [37]. The changes we observed in GR/MR signaling and mitochondrial markers suggest that multiple mechanisms are likely involved in hippocampal atrophy.

We have demonstrated that CCWI leads to significant disruptions in the hippocampus, including altered stress signaling, mitochondrial dysfunction, oxidative damage, and imbalance in neurotrophic proteins such as NFL and RBM3. These changes have been associated with hippocampal atrophy. Vitamin D3 supplementation provides partial protection against oxidative stress and mitochondrial dysfunction. However, it was insufficient to restore all factors to physiological conditions. The increase in RBM3 and NFL content suggests a balance between protective and degenerative processes in the hippocampus. Vitamin D3 may support protective mechanisms, though the effect was limited and insufficient to counteract CCWI’s effects fully. We assume that these results help clarify hippocampal responses to prolonged cold stress and show that supplementation with vitamin D3 **supports the neuroprotective potential** of vitamin D3 in the corticolimbic area and partially attenuates deleterious effects caused by CCWI. However, future studies are necessary to explore multi-modal therapeutic strategies and the role of other molecular and hormonal factors in the adaptive response to cold stress.

## 5. Conclusions

Exposure to CCWI reduced hippocampal mass, induced oxidative damage, mitochondrial dysfunction, and AKT suppression. RBM3 increased, but alone was not enough to counteract neurodegeneration. Although vitamin D3 did not prevent structural changes, it improved mitochondrial enzyme activity and modulated oxidative stress parameters, partially attenuating lipid and protein peroxidation in hippocampal tissue. Even though vitamin D3 supplementation affected some mitochondrial parameters and oxidative stress, it did not prevent the loss of hippocampal mass. Overall, the neuroprotective effect of vitamin D3 in this model remained limited.

### Limitations of the Study

This study has several limitations. The results related to vitamin D3 were obtained in the same cohort of animals as previously described in our study, where systemic and muscle-specific effects were reported [21]. Due to limited hippocampal tissue availability in animals used for analyzed endpoints, Western blot were performed on a reduced number of samples (n = 2–3). In several cases, the number of available samples was too low to allow reliable statistical testing, especially where group differences appeared to follow a trend. Tissue limitations also made it impossible to examine selected downstream proteins in the AKT pathway, including GSK-3β, FOXO, and mTOR, which would have clarified the mechanism responsible for the reduced AKT phosphorylation observed after CCWI. Additionally, behavioral testing was not included in this study. While such data would help relate molecular alterations to hippocampus-dependent functions, the design prioritized biochemical and proteomic endpoints, which required immediate tissue collection following the final cold exposure. As a result, behavioral assessment in the same cohort was not feasible. The absence of these data narrows the interpretive range but reflects the methodological focus of the current study and the limited availability of hippocampal tissue. Despite these constraints, the present results offer a detailed molecular snapshot of the hippocampal response to CCWI and may be useful in guiding further studies linking neurochemical and behavioral endpoints.

## Figures and Tables

**Table 1:** Characteristics of primary and secondary antibodies used for Western blot analysis.

Target Protein	Host Species	Dilution	Catalog Number	Company
GR	Rabbit	1:500	ab183127	Abcam, Cambridge, UK
MR	Rabbit	1:1000	ab64457	Abcam, Cambridge, UK
VDR	Rabbit	1:1000	ab3508	Abcam, Cambridge, UK
COX II	Rabbit	1:1000	NBP294364	Novus Biologicals, Centennial, CO, USA
COX IV	Mouse	1:1000	4D11B3E8	Cell Signaling Technology, Danvers, MA, USA
PGC-1α	Rabbit	1:1000	ab191838	Abcam, Cambridge, UK
BDNF	Rabbit	1:1000	ab108319	Abcam, Cambridge, UK
IGF-1	Rabbit	1:1000	ab9572	Abcam, Cambridge, UK
AKT	Rabbit	1:1000	C67E7	Cell Signaling Technology, Danvers, MA, USA
pAKT	Rabbit	1:1000	D25E6	Cell Signaling Technology, Danvers, MA, USA
NFL	Rabbit	1:1000	C28E10	Cell Signaling Technology, Danvers, MA, USA
RBM3	Rabbit	1:1000	HPA003624	Sigma-Aldrich, St. Louis, MO, USA
**Secondary Antibodies**				
Anti-mouse IgG-HRP	Goat	1:1000–1:5000	ab6728	Abcam, Cambridge, UK
Anti-rabbit IgG-HRP	Goat	1:1000–1:5000	111-035-003	Jackson ImmunoResearch, West Grove, PA, USA

## Data Availability

The datasets used and/or analyzed in the current study are available from the corresponding author upon reasonable request.
